# Impact of group antenatal care (G-ANC) versus individual antenatal care (ANC) on quality of care, ANC attendance and facility-based delivery: A pragmatic cluster-randomized controlled trial in Kenya and Nigeria

**DOI:** 10.1371/journal.pone.0222177

**Published:** 2019-10-02

**Authors:** Lindsay Grenier, Stephanie Suhowatsky, Mark M. Kabue, Lisa M. Noguchi, Diwakar Mohan, Shalmali Radha Karnad, Brenda Onguti, Eunice Omanga, Anthony Gichangi, Jonesmus Wambua, Charles Waka, Jaiyeola Oyetunji, Jeffrey M. Smith

**Affiliations:** 1 Department of Maternal and Newborn Health, Jhpiego, Baltimore, MD, United States of America; 2 Department of Monitoring, Evaluation and Research, Jhpiego, Baltimore, MD, United States of America; 3 Global Epidemiology and Control, Department of International Health, Johns Hopkins Bloomberg School of Public Health, Baltimore, MD, United States of America; 4 Global Programs, Jhpiego, Nairobi, Kenya; 5 Innovations and Technical Leadership, Jhpiego, Nairobi, Kenya; 6 Department of Monitoring, Evaluation, and Research, Jhpiego, Nairobi, Kenya; 7 Global Programs, Jhpiego, Abuja, Nigeria; 8 Technical Leadership Office, Jhpiego, Baltimore, MD, United States of America; Centers for Disease Control and Prevention, UNITED STATES

## Abstract

**Background:**

Low quality and frequency of antenatal care (ANC) are associated with lower uptake of facility-based deliveries—a key intervention to reduce maternal and neonatal mortality. We implemented group ANC (G-ANC), an alternative service delivery model, in Kenya and Nigeria, to assess its impact on quality and attendance at ANC and uptake of facility-based delivery.

**Methods:**

From October 2016‒January 2018, we conducted a facility-based, pragmatic, cluster-randomized controlled trial with 20 clusters per country. We recruited women <24 weeks gestation during their first ANC visit and enrolled women at intervention facilities who agreed to attend G-ANC in lieu of routine individual ANC. The G-ANC model consisted of five monthly 2-hour meetings with clinical assessments alongside structured gestationally specific group discussions and activities. Quality of care was defined as receipt of eight specific ANC interventions. Data were obtained through facility records and self-report during a home-based postpartum survey. Analysis was by intention to treat.

**Findings:**

All women who completed follow up are included in the analysis (Nigeria: 1018/1075 enrolled women [94.7%], Kenya: 826/1013 [81.5%]). In Nigeria women in the intervention arm were more likely to have a facility-based delivery compared to those in the control arm (Nigeria: 76.7% [391/510] versus 54.1% [275/508]; aOR 2.30, CI 1.51–3.49). In both countries women in the intervention arm were more likely than those in the control arm to receive quality ANC (Nigeria: aOR 5.8, CI 1.98–17.21, p<0.001; Kenya: aOR 5.08, CI 2.31–11.16, p<0.001) and to attend at least four ANC visits (Nigeria: aOR 13.30, CI 7.69–22.99, p<0.001; Kenya: aOR 7.12, CI 3.91–12.97, p<0.001).

**Conclusions:**

G-ANC was associated with higher facility-based delivery rates in Nigeria, where those rates associated with individual ANC were low. In both Kenya and Nigeria it was associated with a higher proportion of women receiving quality ANC and higher frequency of ANC visits.

## Introduction

As evidenced in the Sustainable Development Goals [1], ending preventable maternal and neonatal mortality remain global priorities, as do improvements in the overall health and well-being of women and children [2]. Given over 40% of maternal and neonatal deaths occur in the first 24 hours after birth [3], mortality reduction strategies often emphasize skilled intrapartum care and facility-based delivery, still lacking for nearly 31 million births in 2016 [4]. Evidence suggests both the number and quality of antental care (ANC) contacts are associated with facility-based delivery [5–8], as are birth planning and complication readiness (BP/CR) interventions [9–11]. These relationships therefore make ANC a plausible target of efforts to increase facility-based delivery.

ANC, proven to save lives when adequately accessed [12–16], is often an entry point to the health system for women to receive a range of services. Ideally ANC provides health promotion (including birth planning for a facility-based delivery), screening and diagnosis, and disease prevention relevant to gestational age, health status, and geographic context [16, 17]. The *Global Strategy for Women’s*, *Children’s*, *and Adolescents’ Health (2016–2030)*, includes 19 recommended evidence-based interventions in ANC that 1) address major causes of morbidity and mortality, 2) are proven to be highly effective and 3) are critical for the overall health and well-being of women, children, and adolescents; more than during any other category of care [2]. However, ANC can only improve health outcomes if provided with quality, to women who are retained in care.

Since 2001, many low- and middle-income countries (LMICs) have adopted the World Health Organization (WHO) focused ANC model that recommends a minimum of four ANC visits, each of which offers defined gestationally appropriate interventions [18]. However, as measured in the literature by the content of visits and coverage of essential interventions, pregnant women in LMICs continue to receive ANC of inadequate quality [19–21]. In addition to directly impacting the effectiveness of care, low quality ANC is associated with reduced ANC attendance [22, 23]. Globally, while most women now attend at least one ANC visit (86%), only 62% attend four, with lower rates reported in sub-Saharan Africa and South Asia [24].

In 2016, WHO issued *Recommendations on ANC for a Positive Pregnancy Experience*, with an increased emphasis on the experience of care for women and an updated recommendation for ANC models to include a minimum of eight ANC contacts [16]. These recommendations prioritize person-centered care for improved health and well-being [16] and highlight communication and support functions at ANC contacts as key to improving both the quality of care and utilization of health care services. Group ANC (G-ANC), a facility-based alternative service delivery model designed to address these aims, is recommended as a health system intervention in the context of rigorous research [16]. In G-ANC, women of similar gestational ages attend ANC together throughout their pregnancies, actively engaging in their own care [25]. Studies in high-income countries that compare G-ANC to individual ANC show promising but inconsistent results related to increased utilization of care (e.g., attendance at ANC), increased uptake in health practices (e.g., breastfeeding), higher patient satisfaction, and improved health outcomes (e.g., reduced rates of preterm birth) [26, 27]. As a result, G-ANC has been adapted and piloted in a number of LMICs [25]. One study in norther Nigeria has reported a small increase in facility-based delivery among those in G-ANC [28]. Other studies of G-ANC in Africa have not yet reported on the quality of ANC, but have reported significantly higher rates of ANC attendance among women in G-ANC compared to routine individual care [29, 30], and have found G-ANC to be feasible and acceptable to both women and providers [31, 32].

Quality ANC, incorporating BP/CR, has the potential to impact maternal and neonatal health outcomes through increased utilization of facility-based delivery and improved care during pregnancy. This study assessed the effectiveness of G-ANC, compared to individual ANC, in improving rates of facility-based deliveries, ANC attendance and quality of care in two LMIC settings with the intent to compare and contrast observed differences based on country contexts.

## Methods

### Study design and setting

We conducted a facility-based cluster-randomized controlled trial (cRCT) in Nasarawa State, Nigeria, and Kisumu and Machakos Counties in Kenya from October 2016‒January 2018. We chose a pragmatic, parallel cRCT design [33] to gather evidence for the intervention under real-world circumstances in two different African contexts, where we hypothesized some outcomes would be similar while others different, allowing for better understanding of contextual impacts on the model’s outcomes.

Compared to Kenya, Nigeria has lower female literacy (41% versus 74%); contraceptive prevalence (28% versus 61% among women aged 15–49); proportion of pregnant women receiving antenatal care (66% versus 94%); and births assisted by skilled attendants (40% versus 62%) [34]. Conversely, Nigeria has higher fertility and neonatal mortality rates, and a higher maternal mortality ratio than Kenya (5.5 versus 3.8 per woman; 33 vs 21 per 1000 births; and 814 versus 510 per 100,000 births respectively) [34]. Both countries have decentralized health and political administration structures.

Nasawara State is located in the central senatorial zone of Nigeria, Kisumu in western Kenya bordering Lake Victoria, and Machakos in eastern Kenya, bordering Nairobi. All three study areas have areas susceptible to flooding and agriculture is the main economic activity in Nasawara and Machakos. Kisumu has a broader economic base, functioning as a trading hub for western Kenya and the county has a high HIV prevalence (19.3%), associated with the fishing communities of Lake Victoria [35]. Similar to Nasawara, and unlike Machakos, malaria is also endemic in Kisumu.

The unit of randomization was the health facility (a cluster). The study included 20 clusters per country, and individual women were the unit of analysis. In Nigeria, the study was designed in collaboration with the Nasarawa State Ministry of Health (MOH) and Primary Health Care Development Agency. In Kenya, the study was designed with the National MOH and health departments in Kisumu and Machakos Counties. We obtained ethical approval from the Johns Hopkins Bloomberg School of Public Health Institutional Review Board, Nasarawa State MOH, and Kenya Medical Research Institute.

### Participants, randomization, and masking

Study facilities had to have an adequate monthly number of new ANC (ANC1) clients to form gestationally matched cohorts of at least eight women, as well as two or more clinical providers available during ANC clinic hours. Final facility selection considered opportunities for best fit when matching: health facility type/level (representing similar infrastructure; services; and staffing); monthly census of ANC1 clients; urban, periurban, or rural location; culturally similar catchment populations; and availability of a range of family planning services. Matched facility pairs were randomized through a paper-based lottery by staff who had no direct role in study implementation and were not familiar with the individual facilities. Pair by pair these staff blindly chose slips of paper (with facility names) from a basket, assigning the first chosen to intervention, and the remaining to control. Participants, providers, and study staff were not masked to group assignment at any time.

All women attending ANC1 during the enrollment period were screened for eligibility. Participants were required to meet gestational age requirements (ie, ≤24 weeks gestation at time of enrollment in control sites; 20‒24 weeks gestation at time of their assigned group’s first meeting in intervention sites), be ≥15 years old, able to provide a phone number, and have no plans to leave the area for more than 4 consecutive weeks during the pregnancy or for more than three months in the first year postpartum. There were no exclusion criteria for previous or current clinical complications. Parental or guardian consent for pregnant minors 15 years of age and above was waived by all three IRBs as pregnancy confers emancipated status in both countries. All participants provided written informed consent and were enrolled by research staff on the same day as ANC1.

### Procedures

Before enrollment, all intervention and control clusters received equal quantities of ANC-related commodities and clinical supplies to supplement existing stock, and ANC providers participated in a 2-day clinical update. In each intervention cluster three providers also participated in a 5-day G-ANC training. They continued to receive mentoring in G-ANC implementation and facilitation skills by study staff throughout the study. At enrollment, women in the intervention arm were placed in cohorts of eight to 15 women within four weeks gestation of each other and invited to attend five monthly G-ANC meetings as an alternative to individual ANC. Cohorts were of mixed age and parity and not segregated by health status (e.g., HIV+, history of obstetric complication).

G-ANC meetings occurred from October 2016 to October 2017, were approximately 2 hours in duration, and facilitated by nurses, midwives, or community health extension workers (who are trained as ANC providers in Nigeria). Each cohort was assigned two facilitators, with the intent that they co-facilitate all meetings for that cohort. Meetings occurred on the grounds of the intervention clusters and provided 1) private individual clinical assessment and management; 2) participatory, facilitated learning; and 3) peer support. The meeting framework was informed by previous G-ANC models (e.g., CenteringPregnancy®) [36], the American College of Nurse-Midwives Home Based Life-Saving Skills methodology [37], and principles for best practices developed by the Global G-ANC Collaborative [38]. The meeting model included highly facilitative, nonhierarchical, patient-centered participatory approaches with attention to literacy levels and cultural norms.

Providers delivered clinical assessment and management according to national clinical guidelines, with no changes to clinical protocols in either study arm. Beyond the initial 2-day clinical update, neither study arm received mentoring or quality improvement support for clinical decision-making or management. In control sites, providers directed patients to attend ANC per standard country guidelines [39, 40], which largely followed those found in focused ANC as defined by WHO [18]. Intervention sites continued to offer individual ANC to women not enrolled in the study and to study participants as desired or needed (e.g., HIV+ women attended additional individual ANC visits with specialists).

We collected data from enrolled women through a baseline survey at ANC1 and a home-based survey of recently delivered women at 3‒6 weeks postpartum. We extracted additional data from patient-held case notes, facility-based registers, and study-specific registers. All data were directly entered into REDCap^TM^ using tablets.

### Outcomes

The primary outcome was the proportion of women reporting facility-based delivery for the index pregnancy, which was measured by self-report during the postpartum survey. Secondary outcomes included completion of birth-planning components, attendance at four or more ANC visits (ANC4+), attendance and timing of postnatal care (PNC) and quality of ANC. We assessed the completion of individual BP/CR actions through self-report, with a composite measure subsequently calculated for the completion of all seven BP/CR components: identified a facility, made a transportation plan, identified a companion, saved money, agreed on a decision-maker, agreed on an alternate decision maker, and prepared a birth kit. Attendance at ANC was measured and compared by self-report and data extraction; Participants self-reported PNC attendance data. The study defined quality of care as the receipt of eight key interventions during pregnancy: long-lasting insecticidal net (LLIN) provided during ANC, client never ran out of iron and folic acid supplements (IFAS), HIV status known before delivery, syphilis testing completed, three or more doses of intermittent preventive treatment in pregnancy (IPTp) for malaria received, blood pressure recorded at every ANC visit, comprehensive counseling received, and danger signs assessed at all ANC visits. To mitigate unequal effects of stock-outs, interventions included in the analysis were limited to those not requiring supplies/commodities or for which the necessary items were provided by the study.

### Sample size and statistical analysis

The sample size was calculated to detect a 15-percentage point difference in the facility-based delivery rates between the treatment arms. The baseline prevalence of facility delivery was estimated to be 40% in Nasawara, Nigeria and 61% in Kenya based on their most recent DHS estimates [41, 42]. A coefficient of variation between clusters—an intraclass correlation of 0.03—was used to calculate the base sample sizes, which were then adjusted to account for 20% attrition. Statistical power was individually maximized in each country after considering sample-size constraints, including the estimated number of eligible clusters. The resulting power was 80% in Nigeria and 85% in Kenya with a final sample calculation of 538 and 513 women per arm in Nigeria and Kenya, respectively.

The analysis was guided by an intention-to-treat methodology. The effect of the intervention on the outcomes was analyzed using generalized estimating equations with generalized linear models clustering on the facility. We used a multivariable logistic regression model across all outcomes, except the composite adverse event, to calculate adjusted odds ratios (aOR) with 95% confidence intervals (CI). We analyzed the composite adverse event outcome using a Poisson model and present the adjusted incidence rate ratios with 95% CIs. For the Nigeria dataset, we conducted sensitivity analyses by excluding three facilities that did not adhere strictly to the randomization process, producing similar estimates. We therefore present results from the full sample. All analyses were performed using R statistical software (R Core Team, 2017). No independent data monitoring committee was utilized. This trial is registered with Pan African Clinical Trials Registry (PACTR201706002254227), and the study protocol is published, along with study tools [43].

## Results

### Participant characteristics

We selected, matched, and randomized 20 eligible clusters in each country. In Nigeria, evolving security concerns resulted in two sites not following their original allocation and the attrition of one site. We replaced the latter and assigned it the same allocation. All changes occurred before implementation, and sites were not made aware of their original allocation.

**Fig 1:**
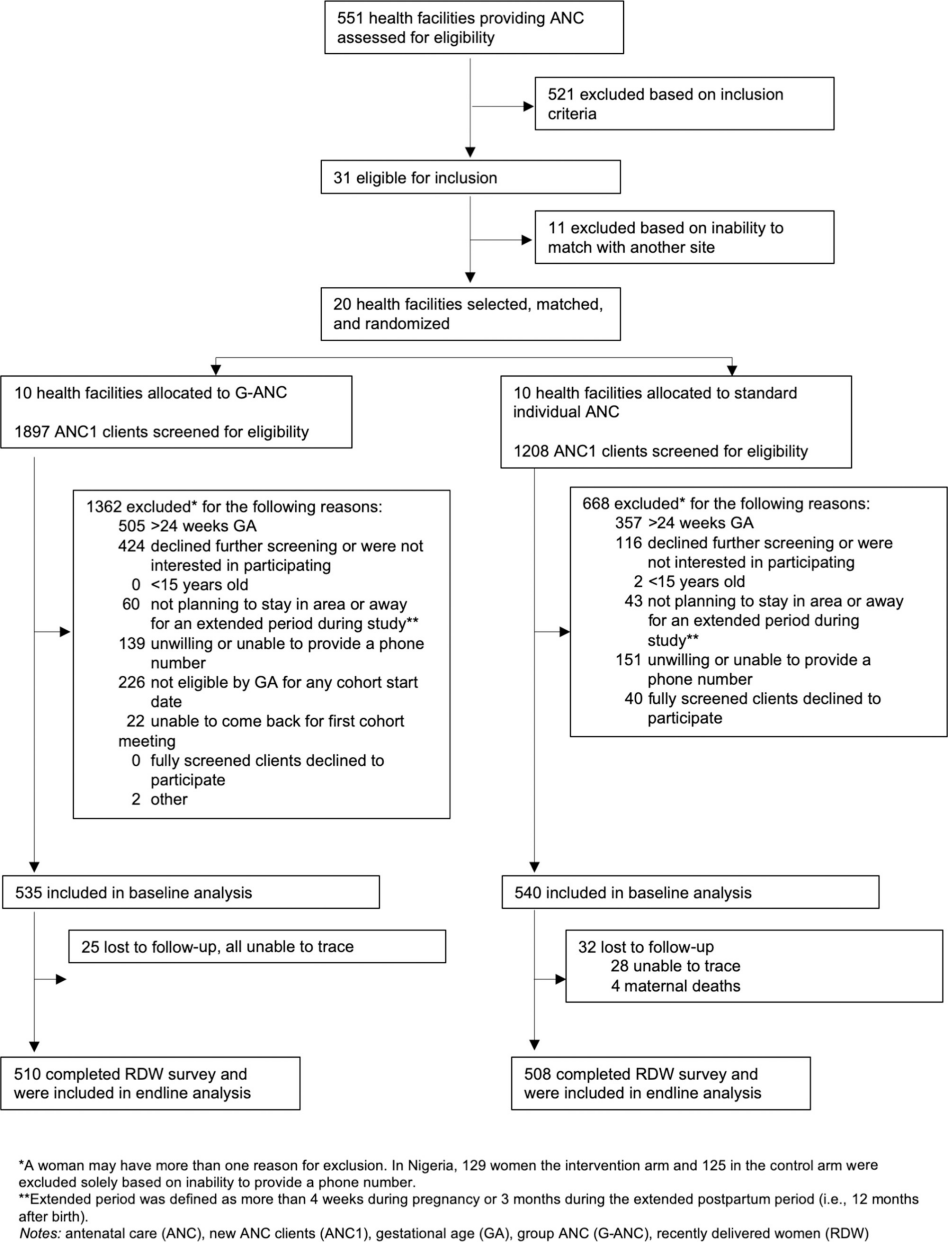
Trial Profile Nigeria.

**Fig 2:**
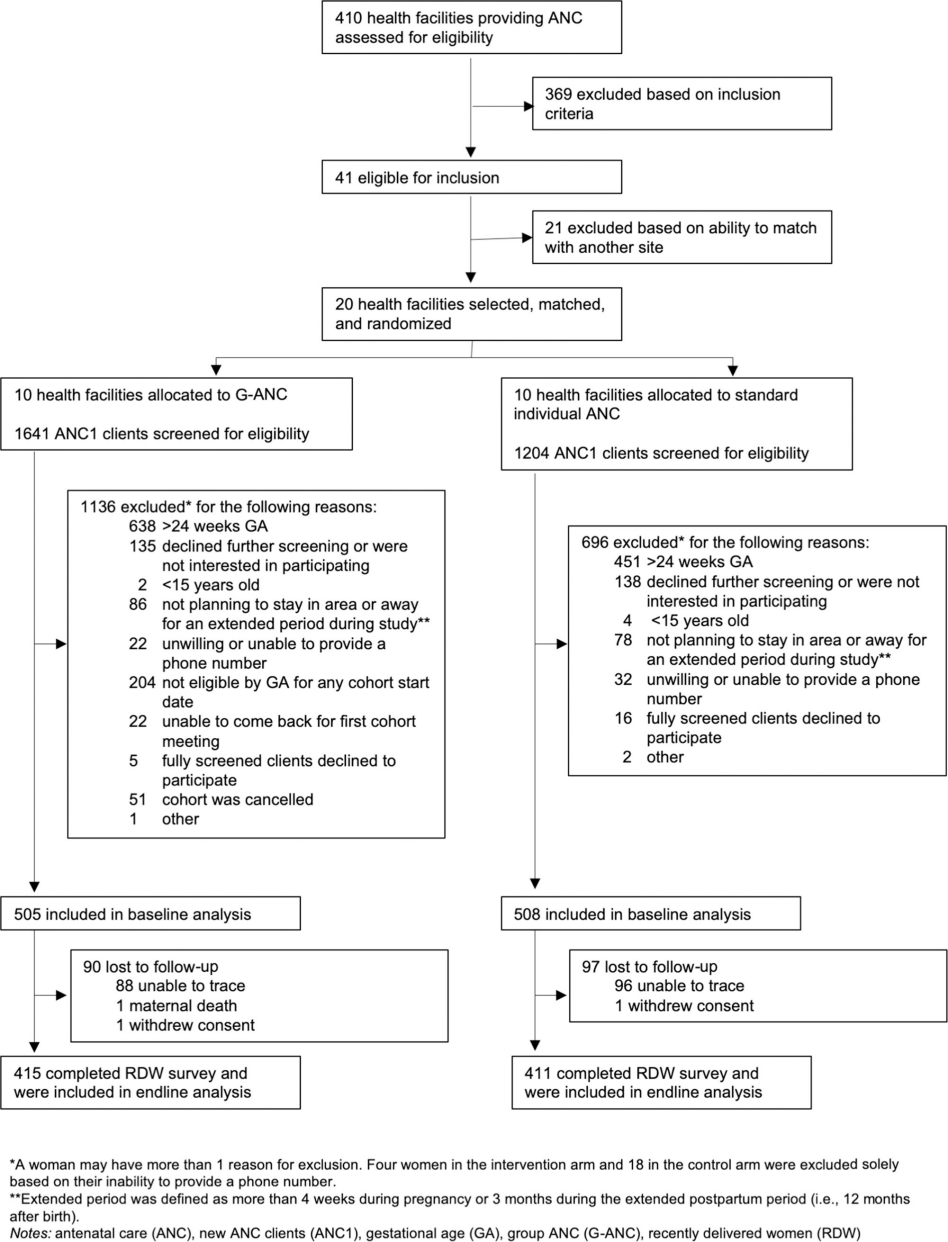
Trial Profile Kenya.

The study enrolled women from October 2016 to June 2017, with even distribution among all clusters. Four or five cohorts were enrolled at each intervention site. Baseline data collection occurred at enrollment, and endline data collection occurred between May 2017 and January 2018. The study excluded 54 women in Kenya from the final sample either due to lack of baseline survey or cancellation of their cohort. Cohort cancellations were primarily due to inadequate enrollment before the start of G-ANC meetings (per protocol), security concerns (related to the 2017 presidential election), and nationwide nursing strikes. In Nigeria, 94.7% (1018/1075) of the participants and in Kenya, 81.5% (826/1013) of the participants completed the postpartum survey; all of whom are included in the primary analysis (Figs 1 and 2).

Compared to Kenya, the study population in Nigeria was older, less educated, more likely to be Muslim, and to be married (Table 1). Of the women enrolled in the intervention arm, 91.4% (466/510) in Nigeria and 87.0% (361/415) in Kenya attended at least one G-ANC meeting. More than three-quarters of these women did not attend any individual ANC visits after ANC1 (Nigeria 379/466; Kenya 278/361; S1 and S2 Figs). After accounting for women who delivered between meetings, the proportion of women who attended each meeting was stable from meeting to meeting (S1 and S2 Figs).

**Table 1:** Demographic characteristics of enrolled women at baseline.

	Nigeria		Kenya*	
	Intervention n = 535 n (%)	Control n = 540 n (%)	Intervention n = 505 n (%)	Control n = 508 n (%)
**Age**				
15–19 years	47 (8.8%)	58 (10.7%)	94 (18.6%)	76 (15.0%)
20–34 years	453 (84.7%)	442 (81.9%)	384 (76.0%)	414 (81.5%)
≥35 years	35 (6.5%)	40 (7.4%)	27 (5.4%)	18 (3.5%)
**Religion (Nigeria)**				
Islam	256 (47.9%)	398 (73.7%)	N/A	N/A
Christian, including Catholic, other	279 (52.1%)	142 (26.3%)	N/A	N/A
**Religion (Kenya)**				
Catholic	N/A	N/A	133 (26.3%)	115 (22.6%)
Other Christian	N/A	N/A	343 (67.9%)	374 (73.6%)
Other	N/A	N/A	29 (5.7%)	19 (3.7%)
**Education**				
No education/primary/Qur’anic cation/Qur’anic	280 (52.3%)	329 (60.9%)	204 (40.4%)	226 (44.5%)
Secondary/postsecondary	255 (47.7%)	211 (39.1%)	301 (59.6%)	282 (55.5%)
**Marriage**				
Never married/single/widowed	9 (1.7%)	2 (0.4%)	79 (15.6%)	91 (17.9%)
Married/cohabiting	526 (98.3%)	538 (99.6%)	425 (84.2%)	417 (82.1%)
**Literacy**				
Cannot read and write	232 (43.4%)	240 (44.4%)	12 (2.4%)	8 (1.6%)
Can read and write	303 (56.6%)	300 (55.6%)	492 (97.4%)	499 (98.2%)
**Parity**				
Zero, never given birth before	166 (31.0%)	152 (28.1%)	204 (40.4%)	175 (34.4%)
>0, never experienced complications	297 (55.5%)	306 (56.7%)	235 (46.5%)	274 (53.9%)
>0, experienced complications	72 (13.5%)	82 (15.2%)	65 (12.9%)	59 (11.6%)
**Facility classification**				
Rural	213 (39.8%)	162 (30.0%)	145 (28.7%)	205 (40.4%)
Urban/periurban	322 (60.2%)	378 (70.0%)	360 (71.3%)	303 (59.6%)
**Mode of transport**				
Walk	206 (38.5%)	238 (44.1%)	191 (37.8%)	206 (40.6%)
Personal	20 (3.7%)	33 (6.1%)	11 (2.2%)	15 (3.0%)
Public	309 (57.8%)	269 (49.8%)	303 (60.0%)	286 (56.3%)
**County**				
Machakos	N/A	N/A	248 (49.1%)	256 (50.4%)
Kisumu	N/A	N/A	257 (50.9%)	252 (49.6%)

*Missing data not shown. Select demographics are missing for two participants in Kenya.

### Facility-Based delivery

Compared to those in the control arm, women in the intervention arm were more likely to deliver in a facility in Nigeria (391/510 [76.7%] versus 275/508 [54.1%]; adjusted OR [aOR] 2.30, CI 1.51–3.49; p<0.001), but not in Kenya where the proportion of facility-based deliveries in both arms was high (348/415 [84.9%] versus 343/411 [83.5%]) (Table 2).

**Table 2:** Effect of G-ANC intervention on service utilization: Facility-based delivery, ANC and PNC.

	Nigeria				Kenya			
	Intervention (n = 510) n(%)	Control (n = 508) n(%)	Adjusted OR (95% CI)*	p value	Intervention (n = 415) n (%)	Control (n = 411) n (%)	Adjusted OR (95% CI)*	p value
**PRIMARY OUTCOME**								
**Facility-based delivery**^†^	391 (76.7)	275 (54.1)	2.30 [1.51, 3.49]	<0.001	348 (83.9)	343 (83.5)	0.92 [0.49, 1.75]	0.810
**SECONDARY OUTCOMES**								
**Antenatal care**								
Attended								
≥4 visits	464 (91.0)	217 (42.3)	13.30 [7.69, 22.99]	<0.001	366 (88.2)	206 (50.1)	7.12 [3.91, 12.97]	<0.001
≥5 visits	418 (82.0)	112 (22.0)	15.06 [9.38, 24.18]	<0.001	294 (70.8)	125 (30.4)	5.86 [3.19, 10.75]	<0.001
≥6 visits	304 (59.6)	57 (11.2)	10.81 [5.87, 19.92]	<0.001	209 (50.4)	50 (12.2)	8.27 [4.32, 15.82]	<0.001
Total # of ANC visits	Median (IQR) 6 (5, 6)	Median (IQR) 3 (2, 5)	N/A	<0.001	Median (IQR) 6 (4, 6)	Median (IQR) 4 (2, 5)	N/A	<0.001
**Postnatal care (PNC)**^†^								
Attended any PNC	336 (65.9)	346 (68.1)	0.75 [0.27, 2.07]	0.572	279 (69.4)	195 (49.4)	2.84 [1.37, 5.90]	0.005
Days from delivery to first PNC visit	Median (IQR) 5 (2, 9)	Median (IQR) 7 (3, 19)	N/A	0.010	Median (IQR) 7 (4, 15)	Median (IQR) 14 (7, 29)	N/A	0.030

* Adjusted for age, religion, education, parity and history of previous complications, and urban or rural location of cluster

^†^ By self-report

In a subgroup analysis of multiparous women in Nigeria, adding location of previous birth to the existing multivariable regression model reduced the effect size but maintained statistical significance (n = 725, aOR 1.93, CI 1.27–2.93; p = 0.002). Among women with a previous home birth, a larger proportion in the intervention versus the control arm switched to a facility-based birth for the index pregnancy. Of women who previously delivered in a facility, a larger proportion in the intervention arm had a repeat facility-based delivery (S1 Table). Including primiparas, we observed similar trends when comparing intended to actual place of delivery (S2 Table).

### Birth planning/Complication readiness

In both countries, women in the intervention arm as compared to the control arm were more likely to complete all seven recommended BP/CR components: identified a facility; made a transportation plan; identified a companion; saved money; agreed on a decision-maker; agreed on an alternate decision maker; and prepared a birth kit to bring to the facility (items as recommended by each facility) (Nigeria: 389/510 [76.3%] versus 193/508 [38.0%]; aOR 4.49, CI 1.52–13.32; p = <0.001; Kenya: 318/415 [76.6%] versus 219/411 [53.3%]; aOR 2.86, CI 1.11–7.38; p = 0.030) (see S3 Table for individual BP/CR component data).

### ANC Attendance

Women in the intervention arms in both countries were significantly more likely than those in the control arms to attend ANC four or more times, with a larger adjusted effect size in Nigeria (aOR 13.30, CI 7.69–22.99) than in Kenya (aOR 7.12, CI 3.91–12.97). We observed a median increase of two ANC visits in Kenya and three in Nigeria (Table 2).

### Postnatal care

In Nigeria, there was no significant difference in attendance at PNC, but median time from delivery to first facility-based PNC visit was 2 days earlier in the intervention versus the control arm (p = 0.010). In Kenya, women in the intervention arm were significantly more likely than women in the control arm to attend at least one PNC visit and attend their first PNC visit earlier—by a median difference of seven days (p = 0.030) (Table 2).

### Quality of care

Women in G-ANC in both countries received higher quality of care, based on the composite measure of ANC quality, with women in the intervention arm more likely to receive all eight ANC interventions compared to those in the control arm (Nigeria: adjusted odds ration [aOR] 5.8, 95% CI 1.98–17.21; p<0.001; Kenya: aOR 5.08, CI 2.31–11.16; p<0.001). Among the eight interventions, the largest effect sizes in both countries were for comprehensive counseling and assessment of danger signs (Table 3. See S4 Table for individual counseling topics and danger signs). In Nigeria, women in the intervention arm were also less likely to ever run out of IFAS and more likely to have received at least three doses of IPTp (Table 3).

**Table 3:** Effects of G-ANC intervention on quality of care.

	Nigeria				Kenya			
	Intervention (n = 510) n(%)	Control (n = 508) n(%)	Adjusted OR (95% CI)*	p value	Intervention (n = 415) n (%)	Control (n = 411) n (%)	Adjusted OR (95% CI)^†^	p value
LLIN provided during ANC	455 (89.2)	505 (99.4)	0.16 [0.02, 1.16]	0.070	391 (94.2)	366 (89.1)	1.80 [0.98, 3.31]	0.056
Never ran out of IFAS tablets^†^	447 (87.6)	357 (70.3)	3.25 [1.31, 8.06]	0.011	295 (71.1)	283 (68.9)	1.14 [0.60, 2.16]	0.697
HIV status known before delivery	507 (99.4)	500 (98.4)	NA	0.170	401 (96.6)	395 (96.1)	1.19 [0.52, 2.74]	0.680
Syphilis testing completed	494 (96.9)	489 (96.3)	NA	0.920	371 (89.4)	381 (92.7)	0.55 [0.16, 1.94]	0.350
IPTP 3+^‡^	347 (68.0)	183 (36.0)	3.80 [1.13, 12.77]	0.031	156 (73.9)	116 (55.8)	2.05 [0.76, 5.53]	0.158
Blood pressure recorded at every ANC visit	462 (90.6)	419 (82.5)	1.82 [0.59, 5.56]	0.296	345 (83.1)	369 (89.8)	0.58 [0.25, 1.34]	0.199
Comprehensive counseling^†^	436 (85.5)	173 (34.1)	8.20 [2.63, 25.71]	<0.001	278 (67.0)	89 (21.7)	7.86 [3.65, 16.92]	<0.001
Assessment of danger signs^†^	459 (90.0)	295 (58.1)	5.67 [2.06, 15.62]	<0.001	362 (87.2)	275 (66.9)	2.87 [1.10, 7.53]	0.032
Received all interventions^§^	220 (43.1)	46 (9.1)	5.80 [1.98, 17.21]	<0.001	134 (32.3)	34 (8.3)	5.08 [2.31, 11.16]	<0.001

* Adjusted for age, religion, education, parity and history of previous complications, and urban or rural location of cluster

^†^ By self-report; all others from data extraction | comprehensive counseling included postpartum family planning options; lactational amenorrhea method; immediate breastfeeding; exclusive breastfeeding; danger signs during pregnancy; care-seeking for danger signs; eating extra food while pregnant and breastfeeding; newborn danger signs; prevention of sexually transmitted infections; use of LLIN; use of IFAS; birth planning | assessment of danger signs included asking woman about pain, fever, bleeding, leakage of fluids, and reduced or no fetal movement at every visit (see S1 Table for data on individual counseling topics and danger signs assessed)

^‡^ IPTp only used in Kisumu County, so percentages represent subjects in that county only: intervention n = 211; control n = 208

^§^ Subjects from Machakos County, Kenya, included if received all but IPTp

*Notes*: odds ratio (OR), confidence interval (CI), interquartile range (IQR), three or more doses of intermittent preventive treatment in pregnancy (IPTp 3+), iron-folic acid supplement (IFAS), long-lasting insecticide-treated net (LLIN), postnatal care (PNC)

## Discussion

In this pragmatic cRCT, women enrolled in G-ANC were more likely to deliver in a facility than women in the control arm in Nigeria, but not in Kenya where facility delivery associated with individual care was equally high. In both countries women enrolled in G-ANC received higher quality ANC care than women in individual ANC, attended more ANC contacts, were more likely to complete all seven BP/CR components, and attended PNC earlier.

This study contributes to the evidence base for G-ANC in LMICs, called for by Catling and colleagues [27] and WHO [16]. In Nigeria, in contrast to the quasi-experimental study of Eluwa et al [28] in Kano State, we found a much larger effect size for facility-based delivery in Nasarawa State, which remained significant after multivariate analysis. This study is the first to report on differences in quality of care, and supports previous findings on increased ANC attendance in comparable resource constrained settings in Malawi, Tanzania, Ghana, and Northern Nigeria [28–30].

Of the eight interventions used to define quality ANC, we found differences in the provision and uptake of services that require repeat visits (e.g., three or more doses of IPTp and comprehensive counseling), but not in those routinely provided in ANC1 (e.g., LLINs and HIV and syphilis testing). This likely reflects high commodity availability and uptake of routine interventions at ANC1 in this study. We hypothesized that G-ANC would only impact receipt of these interventions if initial uptake at the first visit was low. G-ANC may impact the provision and uptake of interventions in subsequent ANC visits in three ways: 1) by increasing ANC attendance; 2) by improving patient willingness to take up interventions; and 3) by reorganizing care, such as including interventions within the meeting structure. For example, providers collected and prepared IPTp supplies ahead of time and administered it to women by direct observed therapy who had already heard and shared information about the purpose of IPTp and had had the opportunity to share and discuss any concerns during structured group discussion at the first meeting.

Studies have previously established an association between the quality and quantity of ANC and facility-based delivery [5–7]. We theorize that the provision and experience of care that G-ANC provides within a cohesive peer group establishes a self-reinforcing cycle that motivates pregnant women to continue care. The stronger this self-reinforcing cycle, the more the pregnant women are exposed to the intervention, which by design provides opportunities to discuss the rationale, experiences, concerns, barriers, and solutions related to BP/CR and facility-based delivery. The improved quality of care and G-ANC design components, such as increased provider-woman contact time and participatory approaches, additionally may lead to improved relationships and trust between pregnant women and their providers. Together, these experiences may contribute to observed changes in both desire for a facility-based delivery and a woman’s ability to act on that desire (S1 and S2 Tables). Future qualitative analyses will report on the experience of G-ANC, including relationships with peers and providers, and compare satisfaction with care between women enrolled in G-ANC and individual ANC. Further development of validated measures for constructs such as experience of care, social capital, trust, empowerment, self-efficacy (specific to facility-based delivery), and agency, are needed to test and refine this theory.

Several factors may explain the difference in the proportion of facility-based deliveries observed between the study arms in Nigeria but not in Kenya. There are likely different causal profiles for out of facility births in contexts with low, medium, and high facility-based delivery rates. Eluwa et al [28], who reported an extremely low proportion of facility-based delivery associated with routine individual care (7.7% [20/260]), failed to find an increase associated with G-ANC after multivariate analysis. The predominant reasons for non-facility-based deliveries may only shift to a mix significantly impacted by exposure to G-ANC when those rates are neither extremely low (as in Eluwa et al, 7.7% [28]), nor relatively high (as in Kenya, 83.5%). Additionally, in Kenya, the intervention effect may have been confounded by other maternal and newborn health programs in the study regions that promoted facility-based delivery (i.e., the Linda Mama national maternity insurance scheme, the Maternal and Child Survival Program in Kisumu County, and APHIAplus Kamili in Machakos County). Lastly, health worker strikes in Kenya during the course of the study and public unrest after national elections caused some disruption to G-ANC meetings and impacted the availability of skilled providers for delivery in public facilities.

### Strengths

This study has several notable strengths, particularly its randomized, prospective, and pragmatic design. Existing facility infrastructure and human resources were used for implementation under real-world settings with similar flexibility in the delivery of G-ANC to what would be anticipated in a non-study setting. Providers, not study staff, carried out the actions that would be necessary for implementation of this service delivery model (e.g., logistics of planning and holding group meetings within the facility, screening by gestational age and explaining G-ANC to prospective participants, facilitating meetings, and calling women to remind them of upcoming meetings). To mimic the majority of settings where ANC census would not allow for the formation of ‘characteristic-specific’ cohorts, G-ANC cohorts were of mixed maternal age and parity. No clinical exclusion criteria were used. In Nigeria, a substantial number of nonliterate women participated (Table 1). In addition, study sites included both urban/periurban and rural catchment areas and different levels of health facilities, thereby increasing the generalizability of findings.

### Limitations

Limitations to our findings include potential sources of bias. Selection bias related to phone access may have been introduced as the inability to provide a phone number was the sole reason for study exclusion in 8.2% of screened clients in Nigeria (n = 254/3105). In Kenya, where cellphone coverage is very high, only 0.8% of screened clients were excluded solely for this reason (n = 22/2805) (Fig 1). The possibility of information bias due to self-reported data for certain outcomes should also be acknowledged. However, where we were able to abstract data in addition to what was provided through self-report, all inferences held—i.e., for receipt of LLINs, ever given IFAS, HIV testing during ANC, and number of ANC visits attended. Study staff completed semi-structured environmental tracking forms during each site visit to record potential sources of bias or confounding. Limited stock-outs of LLINs and IPTp were noted and may have occurred unevenly between the study arms as intervention sites could more easily earmark supplies for study participants. Additionally, as commodity availability impacts quality of care, and previous literature has shown quality impacts attendance [22, 23], results may not be replicable in contexts where key commodities are not available. Furthermore, although here “quality” was defined in terms of intervention coverage, we recognize that this definition represents only a fraction of what constitutes quality care, omitting other critical components such as complication management and patient experience. Study staff also noted that as the study progressed, providers reported that new ANC clients initiating care at intervention sites began to request participation in groups, indicating that self-selection bias may have strengthened as the study progressed. In Kenya, the availability of ANC services in intervention and control arms was disrupted by four national health care worker strikes. With few exceptions, in intervention facilities, facilitators continued to offer G-ANC, while in control facilities, individual ANC services were sometimes unavailable. In Nigeria, both intervention and control sites participated in a concurrent World Bank performance-based financing program that rewarded facility-based delivery. This may have provided a synergistic effect, enhancing the observed effect size for facility-based delivery and G-ANC.

We are unable to comment on G-ANC’s potential relationship to pregnancy loss, infant mortality, and maternal mortality due to lack of study power. We include frequencies for these data in S5 Table.

### Considerations for implementation

Findings from this study support wider implementation of G-ANC in LMICs as one strategy to meet WHO’s ambitious eight ANC contact target while simultaneously improving the quality of ANC and facility-based delivery (where low). However, generalizability of findings to other locations requires consideration of several factors: frequency of health service disruptions, freedom of movement of the pregnant population, average gestation at entry to ANC and monthly census of women attending ANC1, availability of key ANC commodities, availability of additional staff to attend to non-G-ANC clients during meetings, availability and quality of G-ANC mentorship, and access to mobile phones and airtime by both G-ANC participants and providers (increasingly available in many low-resource settings).

## Conclusion

G-ANC research in high-income countries, where G-ANC was initially developed and where the preponderance of its implementation has been, has often excluded salient outcomes of interest to LMICs. Compelling reasons to implement G-ANC in LMICs may thus not be captured in systematic reviews based on studies in high-income countries. Our results, from a pragmatic study, suggest that a G-ANC model purposefully built for the context, which integrates social and behavioral change and focuses on issues common to LMICs, can improve the quality of and attendance at facility-based ANC as well as raise low facility-based delivery rates. Given the lack of harm in implementing G-ANC—as consistently found in previous studies—these results support wider implementation and evaluation of G-ANC by LMICs looking to improve these outcomes.

### Recommendations

For a new service delivery model to have a transformational influence on maternal health care, it needs to be available to a large portion of the population. As such, future research should explore both the feasibility and impact of G-ANC adaptations in a variety of contexts (e.g., large busy tertiary hospitals, private facilities, facilities with low ANC census) and report on the percentage of clients served through the group model. Additional exploration of G-ANC’s effect on facility-based delivery in contexts with varying baseline rates is warranted alongside further research to identify: if availability of G-ANC incentivizes earlier entry to care, if G-ANC participation influences choice of facility for routine facility-based delivery (i.e., at same site where ANC was received), how G-ANC impacts delivery of other services (positively or negatively), and how G-ANC can improve family engagement in care. In addition, future studies should be undertaken and reported with careful recognition that both specifics of the individual model being tested and implementation quality may alter results. As recommended by the Global G-ANC Collaborative, careful reporting of intervention components (e.g., number, structure, and content of meetings; designation and training of facilitators; mentoring of new sites) will aid interpretation and the ability to identify best practices, as well as potential aggregation of data from different studies [38]. Additional data on clinical outcomes are also still needed, ideally incorporating outcomes from subsequent births to capture potential gains related to G-ANC participation in the index birth. Finally, if G-ANC is to provide a substantial, sustained benefit, there must be a continued commitment to widespread health system strengthening. While G-ANC may be a successful strategy for improving service delivery, that success is interdependent with strengthening the other five health system building blocks as outlined by WHO: health workforce, health information systems, access to essential medicines, financing, and leadership/governance [44].

## Supporting information

S1 Fig Exposure to intervention, Nigeria: (A) G-ANC attendance by meeting and (B) type(s) of ANC attended after first ANC visit.
(PDF)

S2 Fig Exposure to intervention, Kenya: (A) G-ANC attendance by meeting and (B) type(s) of ANC attended after first ANC visit.
(PDF)

S1 Table Location of current delivery compared to previous delivery by study group in Nigeria, multiparas only. (DOCX)

S2 Table Location of current delivery compared to intent at entry to antenatal care by study group in Nigeria, all subjects. (DOCX)

S3 Table Effect of G-ANC on individual components of birth planning and complication readiness. (DOCX)

S4 Table Effect of intervention on individual components of comprehensive counseling received and danger signs assessed. (DOCX)

S5 Table Pregnancy loss and mortality by study arm. (DOCX)

S1 Data G-ANC data codebook. (CSV)

S2 Data G-ANC data, Kenya and Nigeria. (ZIP)

S1 Checklist CONSORT checklist. (PDF)
