# Supplementary material for: Impact of group antenatal care (G-ANC) versus individual antenatal care (ANC) on quality of care, ANC attendance and facility-based delivery: A pragmatic cluster-randomized controlled trial in Kenya and Nigeria
Source: PLoS One. 2019 Oct 2;14(10):e0222177. doi: 10.1371/journal.pone.0222177 (PMC6774470; doi:10.1371/journal.pone.0222177)
Supplement: S2 Fig — Exposure to intervention, Kenya: (A) G-ANC attendance by meeting and (B) type(s) of ANC attended after first ANC visit. (PDF) [file pone.0222177.s002.pdf]

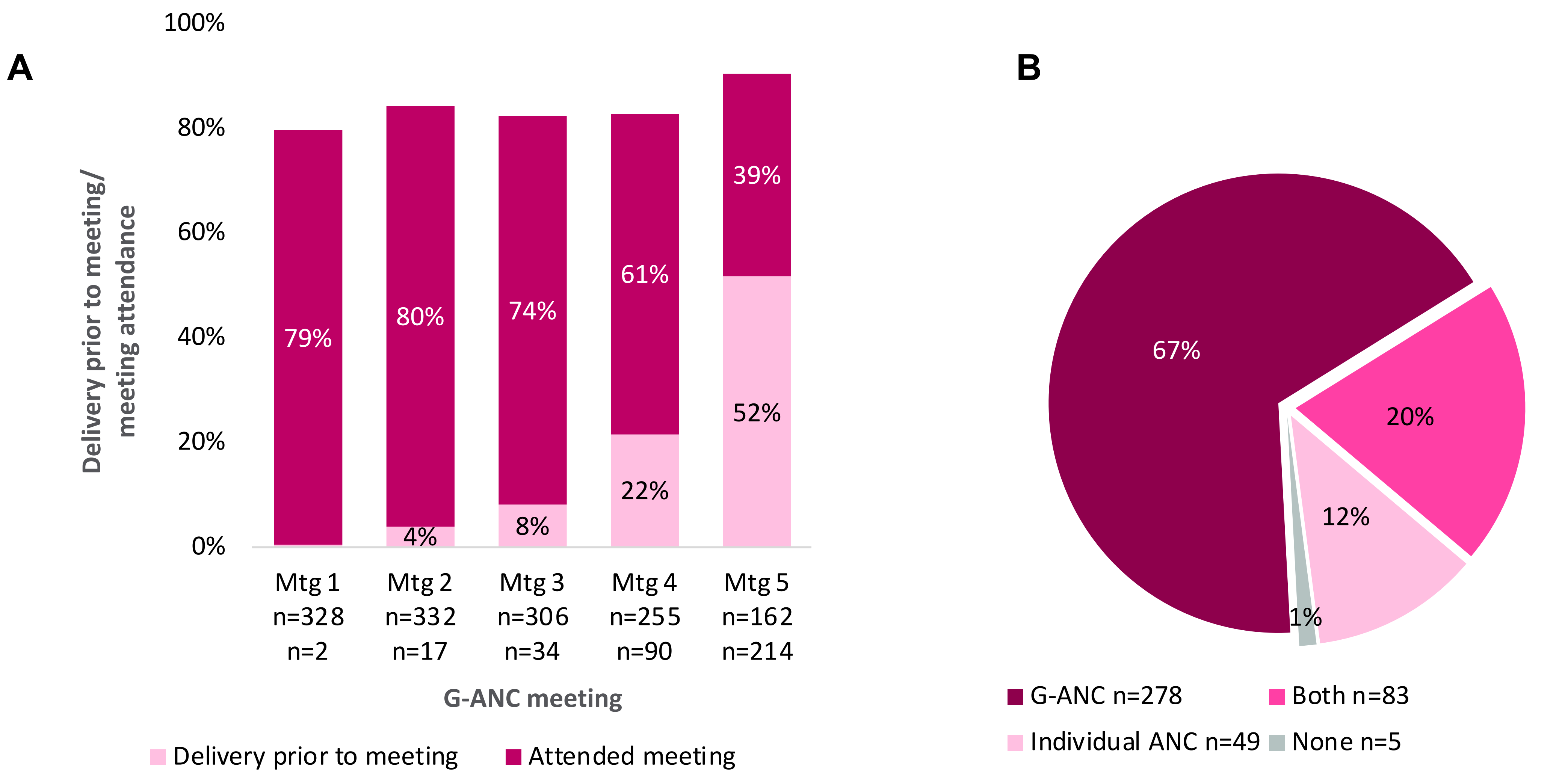

(A) Proportion of women who delivered prior to each meeting and proportion who attended each meeting. Women may have returned to meetings after delivering. (B) Type of ANC attended after first ANC visit.

Notes: antenatal care (ANC), group ANC (G-ANC), meeting (Mtg)

Supplemental Figure 2: **Exposure to intervention in Kenya (n=415)**
