# Supplementary material for: Impact of group antenatal care (G-ANC) versus individual antenatal care (ANC) on quality of care, ANC attendance and facility-based delivery: A pragmatic cluster-randomized controlled trial in Kenya and Nigeria
Source: PLoS One. 2019 Oct 2;14(10):e0222177. doi: 10.1371/journal.pone.0222177 (PMC6774470; doi:10.1371/journal.pone.0222177)
Supplement: S1 Table — (DOCX) [file pone.0222177.s003.docx]

**S1 Table: Location of current delivery compared to previous delivery by study group in Nigeria, multiparas only**

|  | **Location of current delivery** | | | | | | | |
| --- | --- | --- | --- | --- | --- | --- | --- | --- |
| **Location of previous delivery** | **Intervention**  **n=351**  **n (%)** | | | | **Control**  **n=361**  **n (%)** | | | |
|  | **At home** | **Health facility** | **In transit** | **Total** | **At home** | **Health facility** | **In transit** | **Total** |
| At home | 48 (32.0) | 103 (68.0) | 0 (0.0) | 150 (100.0) | 113 (63.5) | 59 (33.1) | 6 (3.4) | 178 (100.0) |
| At health facility | 39 (19.5) | 158 (79.0) | 3 (1.5) | 200 (100.0) | 61 (34.1) | 116 (64.8) | 2 (1.1) | 179 (100.0) |
| In transit | 0 (0.0) | 1 (100.0) | 0 (0.0) | 1 (100.0) | 0 (0.0) | 3 (75.0) | 1 (25.0) | 4 (100.0) |
| **Total** | 87 (24.8) | 261 (74.4) | 3 (0.0) | 351 (100.0) | 174 (48.2) | 178 (49.3) | 9 (2.5) | 361 (100.0) |
