# Supplementary material for: Impact of group antenatal care (G-ANC) versus individual antenatal care (ANC) on quality of care, ANC attendance and facility-based delivery: A pragmatic cluster-randomized controlled trial in Kenya and Nigeria
Source: PLoS One. 2019 Oct 2;14(10):e0222177. doi: 10.1371/journal.pone.0222177 (PMC6774470; doi:10.1371/journal.pone.0222177)
Supplement: S2 Table — (DOCX) [file pone.0222177.s004.docx]

**S2 Table:** **Location of current delivery compared to intent at entry to antenatal care by study group in Nigeria, all subjects**

|  | **Location of current delivery** | | | | | | | |
| --- | --- | --- | --- | --- | --- | --- | --- | --- |
| **Delivery location intention at entry to ANC** | **Intervention**  **n=504**  **n (%)** | | | | **Control**  **n= 502**  **n (%)** | | | |
|  | **At home** | **Health facility** | **In transit** | **Total** | **At home** | **Health facility** | **In transit** | **Total** |
| At home | 18 (37.5) | 29 (60.4) | 1 (2.1) | 48 (100.0) | 60 (72.3) | 22 (26.5) | 1 (1.2) | 83 (100.0) |
| At health facility | 67 (18.1) | 301 (81.4) | 2 (0.5) | 370 (100.0) | 110 (34.3) | 207 (64.5) | 4 (1.2) | 321 (100.0) |
| Not sure/ undecided | 25 (29.1) | 61 (70.9) | 0 (0.0) | 86 (100.0) | 45 (46.4) | 46 (47.4) | 7 (7.2) | 98 (100.0) |
| **Total** | 110 (21.8) | 391 (77.6) | 3 (0.6) | 504 (100.0) | 215 (42.8) | 274 (54.8) | 12 (2.4) | 502(100.0) |
