# Supplementary material for: Impact of group antenatal care (G-ANC) versus individual antenatal care (ANC) on quality of care, ANC attendance and facility-based delivery: A pragmatic cluster-randomized controlled trial in Kenya and Nigeria
Source: PLoS One. 2019 Oct 2;14(10):e0222177. doi: 10.1371/journal.pone.0222177 (PMC6774470; doi:10.1371/journal.pone.0222177)
Supplement: S3 Table — (DOCX) [file pone.0222177.s005.docx]

**S3 Table: Effect of G-ANC on individual components of birth planning and complication readiness**

|  | **Nigeria** | | | | **Kenya** | | | |
| --- | --- | --- | --- | --- | --- | --- | --- | --- |
|  | **Intervention (n=510)**  **n (%)** | **Control (n=508)**  **n (%)** | **Adjusted**  **OR (95% CI)*** | **p value** | **Intervention (n=415)**  **n (%)** | **Control (n=411)**  **n (%)** | **Adjusted**  **OR (95% CI)*** | **p value** |
| **Birth planning** |  |  |  |  |  |  |  |  |
| Identified a facility | 475 (93.1) | 355 (69.9) | 4.73 [2.27, 9.74] | <0.001 | 385 (92.8) | 348 (84.7) | 2.21 [1.16, 4.20] | 0.016 |
| Made transportation plan | 457 (89.6) | 323 (63.6) | 4.23 [1.77, 10.12] | 0.001 | 385 (92.8) | 336 (81.8) | 2.70 [1.22, 5.94] | 0.014 |
| Identified companion | 462 (90.6) | 352 (69.3) | 3.44 [1.71, 6.89] | <0.001 | 382 (92.0) | 311 (75.7) | 4.03 [1.79, 9.08] | <0.001 |
| Saved money | 477 (93.5) | 407 (80.1) | 3.27 [1.48, 7.23] | 0.003 | 380 (91.6) | 345 (83.9) | 2.04 [0.85, 4.86] | 0.108 |
| Agreed on decision-maker | 468 (91.8) | 378 (74.4) | 2.91 [1.19, 7.10] | 0.019 | 382 (92.0) | 312 (75.9) | 4.05 [1.52, 10.80] | 0.005 |
| Agreed on alternate decision-maker | 405 (79.4) | 262 (51.6) | 2.77 [1.00, 7.69] | 0.050 | 339 (81.7) | 273 (66.4) | 2.02 [0.75, 5.47] | 0.170 |
| Prepared a birth kit | 490 (96.1) | 392 (77.2) | 11.67 [3.38, 40.30] | <0.001 | 395 (95.2) | 360 (87.6) | 3.03 [1.01, 9.10] | 0.048 |
| **Took all birth planning actions** | 389 (76.3) | 193 (38.0) | 4.49 [1.52, 13.32] | <0.001 | 318 (76.6) | 219 (53.3) | 2.86 [1.11, 7.38] | 0.030 |

* Adjusted for age, religion, education, parity and history of previous complications, and urban or rural location of cluster

*Notes:* odds ratio (OR), confidence interval (CI)
