# Supplementary material for: Impact of group antenatal care (G-ANC) versus individual antenatal care (ANC) on quality of care, ANC attendance and facility-based delivery: A pragmatic cluster-randomized controlled trial in Kenya and Nigeria
Source: PLoS One. 2019 Oct 2;14(10):e0222177. doi: 10.1371/journal.pone.0222177 (PMC6774470; doi:10.1371/journal.pone.0222177)
Supplement: S4 Table — (DOCX) [file pone.0222177.s006.docx]

**S4 Table: Effect of intervention on individual components of comprehensive counseling received and danger signs assessed**

|  | **Nigeria** | | | **Kenya** | | |
| --- | --- | --- | --- | --- | --- | --- |
|  | **Intervention**  **n=510**  **n (%)** | **Control**  **n=508**  **n (%)** | **p value** | **Intervention**  **n=415**  **n (%)** | **Control**  **n=411**  **n (%)** | **p value** |
| **Individual counseling topics: When you went for antenatal care, did a health care provider talk with you about…? (YES shown)** | | | | | | |
| Options for family planning/child spacing | 483 (94.9) | 376 (74.0) | <0.001 | 370 (89.2) | 201 (48.9) | <0.001 |
| How to use breastfeeding to prevent another pregnancy right away | 469 (92.0) | 272 (53.5) | <0.001 | 332 (80.0) | 201 (48.9) | <0.001 |
| When to begin breastfeeding | 482 (94.5) | 371 (73.0) | <0.001 | 367 (88.4) | 299 (72.7) | 0.002 |
| When to introduce other fluids and foods to your baby | 481 (94.3) | 391 (77.0) | <0.001 | 389 (93.7) | 326 (79.3) | <0.001 |
| Danger signs or warning signs to watch for during pregnancy | 484 (94.0) | 304 (59.8) | <0.001 | 386 (93.0) | 303 (73.7) | <0.001 |
| Returning to the clinic if you noticed any danger signs, warning signs, or had other problems | 503 (98.6) | 459 (90.4) | <0.001 | 391 (94.2) | 367 (89.3) | 0.190 |
| Eating extra food while pregnant and breastfeeding | 498 (97.6) | 455 (89.6) | <0.001 | 389 (93.7) | 322 (78.3) | 0.037 |
| Danger signs or problems to watch out for in your newborn | 474 (92.9) | 285 (56.1) | <0.001 | 365 (88.0) | 289 (70.3) | <0.001 |
| How to prevent transmission of sexually transmitted infections? | 473 (92.7) | 305 (60.0) | <0.001 | 378 (91.1) | 239 (58.2) | <0.001 |
| The importance of sleeping under a bed net | 500 (98.0) | 481 (94.7) | 0.037 | 409 (98.6) | 379 (92.2) | 0.010 |
| The importance of taking iron and folic acid tablets | 492 (96.5) | 392 (77.2) | 0.001 | 393 (94.7) | 352 (85.6) | 0.025 |
| The importance of making a birth plan and what to plan for | 476 (93.3) | 353 (69.5) | 0.013 | 386 (93.0) | 291 (70.8) | <0.001 |
| All 12 topics covered | 436 (85.5) | 173 (34.1) | <0.001 | 278 (67.0) | 89 (21.7) | <0.001 |
| **Individual danger signs assessed: At every antenatal visit, did your provider ask if you had…? (YES shown)** | | | | | | |
| Pain | 499 (97.8) | 433 (85.2) | <0.001 | 397 (95.7) | 360 (87.6) | 0.010 |
| Fever | 488 (95.7) | 393 (77.2) | 0.004 | 382 (92.0) | 328 (79.8) | 0.065 |
| Bleeding | 481 (94.3) | 353 (69.5) | <0.001 | 386 (93.0) | 307 (74.7) | 0.004 |
| Leakage of fluids | 473 (92.7) | 325 (64.0) | 0.001 | 382 (92.0) | 314 (76.4) | 0.007 |
| Reduced or no fetal movement | 494 (96.9) | 453 (89.2) | <0.001 | 403 (97.1) | 388 (94.4) | 0.150 |
| Report all five signs assessed at every visit | 459 (90.0) | 295 (58.1) | <0.001 | 362 (87.2) | 275 (66.9) | 0.022 |
