# Supplementary material for: Impact of group antenatal care (G-ANC) versus individual antenatal care (ANC) on quality of care, ANC attendance and facility-based delivery: A pragmatic cluster-randomized controlled trial in Kenya and Nigeria
Source: PLoS One. 2019 Oct 2;14(10):e0222177. doi: 10.1371/journal.pone.0222177 (PMC6774470; doi:10.1371/journal.pone.0222177)
Supplement: S5 Table — (DOCX) [file pone.0222177.s007.docx]

**S5 Table: Pregnancy loss and mortality by study arm**

|  | **Nigeria** | | **Kenya** | |
| --- | --- | --- | --- | --- |
|  | **Intervention (n=510)**  **n (%)** | **Control (n=508)**  **n (%)** | **Intervention (n=415)**  **n (%)** | **Control**  **(n=411)**  **n (%)** |
| Infant death**^*^** **^†^** | 13 (2.5) | 12 (2.4) | 7 (1.7) | 12 (2.9) |
| Pregnancy loss, including stillbirth^‡^ | 32 (6.3) | 44 (8.7) | 21 (5.1) | 21 (5.1) |
| Maternal death**^*^** | 0 | 4 | 1 | 0 |
| Composite mortality | 45 (8.8) | 60 (11.8) | 29 (7.0) | 33 (8.0) |

**^*^**Up to time of postpartum survey, data collection ranged from 3 weeks postpartum to beyond the neonatal period. Maternal deaths were identified through tracing efforts of the survey.

**^†^** By self-report
